# The Deubiquitinase USP17 Regulates the Stability and Nuclear Function of IL-33

**DOI:** 10.3390/ijms161126063

**Published:** 2015-11-24

**Authors:** Yingmeng Ni, Lianqin Tao, Chen Chen, Huihui Song, Zhiyuan Li, Yayi Gao, Jia Nie, Miranda Piccioni, Guochao Shi, Bin Li

**Affiliations:** 1 Department of Pulmonary Medicine, Ruijin Hospital, Shanghai Jiao Tong University School of Medicine, Shanghai 200025, China; danielle_mengmeng@hotmail.com (Y.N.); tlq.1988@163.com (L.T.); shh_2012@hotmail.com (H.S.); 2 Key Laboratory of Molecular Virology and Immunology, Unit of Molecular Immunology, Institut Pasteur of Shanghai, Shanghai Institutes for Biological Sciences, Chinese Academy of Sciences, Shanghai 200031, China; chenchen01@sibs.ac.cn (C.C.); lizhiyuan01@gmail.com (Z.L.); yygao@sibs.ac.cn (Y.G.); niejia@sibs.ac.cn (J.N.); pimiri@inwind.it (M.P.)

**Keywords:** IL-33, ubiquitin-specific protease 17 (USP17), IL-13, DNA binding, deubiquitinase

## Abstract

IL-33 is a new member of the IL-1 family cytokines, which is expressed by different types of immune cells and non-immune cells. IL-33 is constitutively expressed in the nucleus, where it can act as a transcriptional regulator. So far, no direct target for nuclear IL-33 has been identified, and the regulation of IL-33 nuclear function remains largely unclear. Here, we report that the transcription of type 2 inflammatory cytokine IL-13 is positively regulated by nuclear IL-33. IL-33 can directly bind to the conserved non-coding sequence (CNS) before the translation initiation site in the *IL13* gene locus. Moreover, IL-33 nuclear function and stability are regulated by the enzyme ubiquitin-specific protease 17 (USP17) through deubiquitination of IL-33 both at the K48 and at the K63 sites. Our data suggest that *IL13* gene transcription can be directly activated by nuclear IL-33, which is negatively regulated by the deubiquitinase USP17.

## 1. Introduction

Interleukin-33 (IL-33) was initially discovered as a nuclear factor in endothelial cells and named for this reason NF-HEV (nuclear factor from high endothelial venules) [1]. Afterwards, it was identified as a novel member of the IL-1 cytokine family, binding to the orphan IL-1 receptor family member ST2 (also known as IL-1RL1) [2]. IL-33 is constitutively expressed by non-immune cells, including epithelial cells, fibroblasts and endothelial cells, as well as innate immune cells, including dendritic cells and macrophages [3]. Under normal conditions, IL-33 is stored in the nucleus acting as a transcription factor, while upon cellular stress or damage (such as infection and injury), it can be released in the extracellular space through cellular necrosis or unconventional secretory mechanisms and function as alarmin [4]. Once released, IL-33 activates various types of cells, such as basophils, mast cells, eosinophils, macrophages, dendritic cells, T helper 2 lymphocytes, natural killer (NK) and natural killer T (NKT) cells, by binding to its receptor complex consisting of ST2 and the IL-1 receptor accessory protein (IL-1RAcP). The binding of IL-33 to the ST2 receptor elicits the activation of the NF-κB and MAP kinases pathways, finally leading to immune activation [5]. As a cytokine, extracellular IL-33 is closely involved in Th2 immune responses in allergic and autoimmune diseases and plays a role in the host defense against infection. As a nuclear factor, IL-33 is involved in the regulation of transcription. Indeed, full length IL-33 contains an evolutionarily-conserved homeodomain-like helix-turn-helix (HTH) DNA binding domain in the N-terminus, which is necessary for interacting with heterochromatin and mitotic chromatin [6,7]. Although its structure is already known, the functional role and regulation of nuclear IL-33 remain unclear. A recent study suggests that the binding of IL-33 to the NF-κB p65 subunit in the nucleus could reduce p65-triggered gene expression to dampen the production of proinflammatory cytokines [8]. However, another study also suggests that nuclear IL-33 could bind to the promoter region of *p65*, positively regulating its transcription in endothelial cells [9].

Our study has showed the existence of ubiquitination of IL-33, which can be regulated by deubiquitinase in human embryonic kidney (HEK) 293T cells [10]. Ubiquitination, together with other post-translational modifications (PTMs), plays critical roles in regulating various cellular processes [11]. Ubiquitination is mediated by seven different lysines (K) (K6, K11, K27, K29, K33, K48 and K63) and leads either to proteasomal degradation or to the modulation of protein activity [12,13]. For example, it is well known that K48-linked ubiquitination contributes to the proteasomal degradation of many target proteins [14], while monoubiquitination or K63-linked polyubiquitination exerts several new functions, including activation of enzymatic proteins, control of gene transcription, DNA repair and vesicle trafficking, as well [15,16]. Thus, we wondered if the ubiquitination of IL-33 could also regulate the nuclear function of IL-33, while maintaining its stability.

Ubiquitin modification can be reversed by a large family of enzymes termed deubiquitinases (DUBs). There are mainly five families of DUBs: ubiquitin C-terminal hydrolases (UCHs), ubiquitin-specific proteases (USPs), ovarian tumor proteases (OTUs), Josephins and JAB1/MPN/MOV34 metalloenzymes (JAMMs) [17]. USPs, which constitute the largest family with more than 60 members, are emerging as potential target sites for pharmacological interference of the ubiquitin regulatory network [18]. The expression of deubiquitinase USP17 could be induced by inflammatory cytokines, such as IL-4 and IL-6 [19], which is responsible for the regulation of cell growth and survival [20] and regulates cell proliferation by reversing K63-linked ubiquitination of RCE1 [21]. USP17-mediated K63-linked deubiquitination can also regulate the biological functions of SDS3, resulting in blocking histone deacetylase (HDAC) activity in cancer cells [22]. A recent study has shown that USP17 can interact with Cdc25A and remove the K48-linked polyubiquitin chains, which target Cdc25A for proteasomal degradation, in order to subvert cell cycle and promote tumor progression [23]. Therefore, USP17 can regulate both K48 and K63-linked polyubiquitination of its target proteins to modulate their stability and/or activity.

Here, we identified a post-translational modification of IL-33 by lysine polyubiquitination regulated by the deubiquitinase USP17. USP17 could deubiquitinate both K48 and K63-linked polyubiquitination of IL-33, then regulate its stability and nuclear function.

## 2. Results

### 2.1. IL-33 Can Be Modified by Protein Polyubiquitination

**Figure 1:**
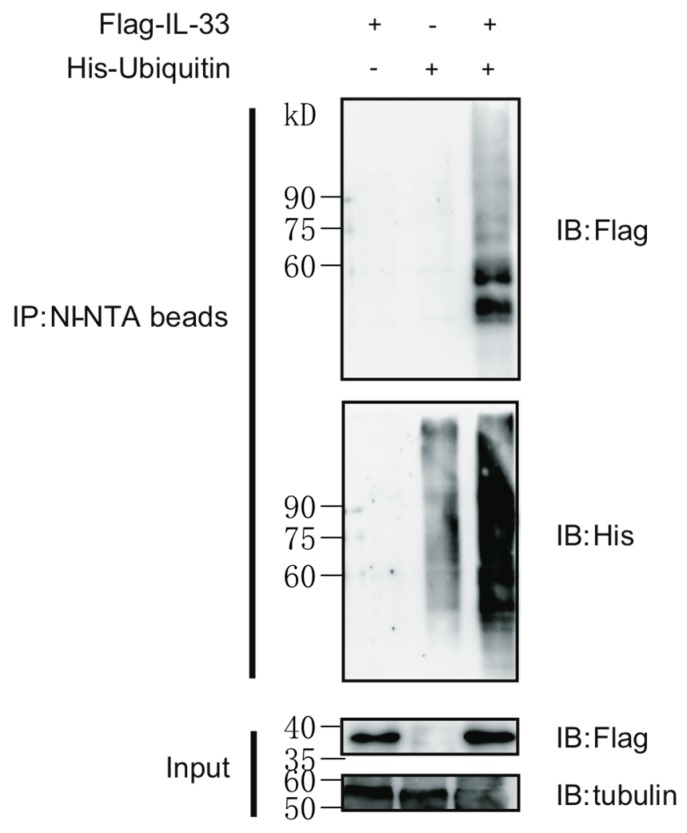
IL-33 can be modified by polyubiquitination. HEK293T cells were transfected with the plasmids of Flag-tagged IL-33 and/or His-tagged ubiquitin. Cells were treated with 20 nM MG132 for 4 h before harvesting. Ubiquitinated IL-33 was pulled-down with Ni-NTA beads upon denaturing conditions as described in the Materials and Methods. Immune blots (IB) were performed with indicated antibodies. Data are representative of at least three independent experiments.

Post-translational modifications (PTMs) are of pivotal importance in regulating the function of many cellular proteins. Ubiquitination allows proteins to be tagged and recognized by the ubiquitin-proteasome system in order to be degraded. We firstly detected the ubiquitination status of IL-33 under ectopic expression. To this aim, expression vectors encoding Flag-tagged IL-33 and His-tagged ubiquitin were co-transfected into human embryonic kidney (HEK) 293T cells for a ubiquitin pull-down assay. The polyubiquitinated IL-33 was pulled down by Ni-NTA beads and detected by immune blot (Figure 1). Our data suggest that IL-33 can be post-translationally modified by lysine polyubiquitination.

### 2.2. Ubiquitin-Specific Protease 17 (USP17) Associates with IL-33

**Figure 2:**
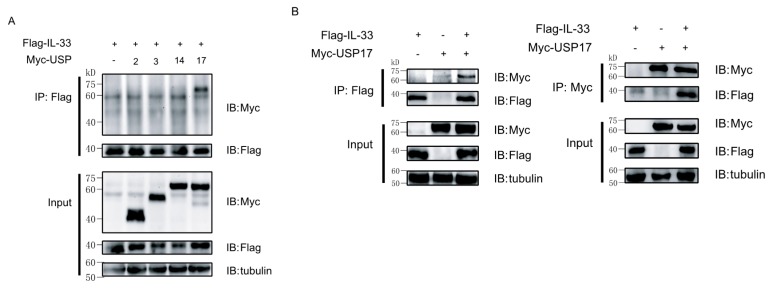
The deubiquitinase USP17 interacts with IL-33. (**A**) HEK293T cells were transfected with plasmids encoding Flag-IL-33 and several ubiquitin-specific proteases (USPs) or an empty vector as a negative control. Immunoprecipitation (IP) was performed with anti-Flag antibody, and immune blots (IB) were then performed with the indicated antibodies; (**B**) HEK293T cells were transfected with Flag-IL-33 and/or Myc-USP17. Immunoprecipitation (IP) was performed with either anti-Flag antibody or anti-Myc antibody. Immune blots were performed with the indicated antibodies. Data are representative of at least three independent experiments.

Since IL-33 can be modified by polyubiquitination, we then screened potential DUBs, which could interact with IL-33. For this reason, Myc-tagged USP2, USP3, USP4 and USP17 and Flag-tagged IL-33 were co-transfected into 293T cells for a co-immunoprecipitation assay. The results revealed that USP17 was the main DUB interacting with IL-33 (Figure 2A). Then, we performed a reciprocal immunoprecipitation assay by co-transfecting Myc-tagged USP17 and Flag-tagged IL-33 into 293T cells to confirm the interaction between IL-33 and USP17 (Figure 2B).

### 2.3. USP17 Deubiquitinates IL-33

**Figure 3:**
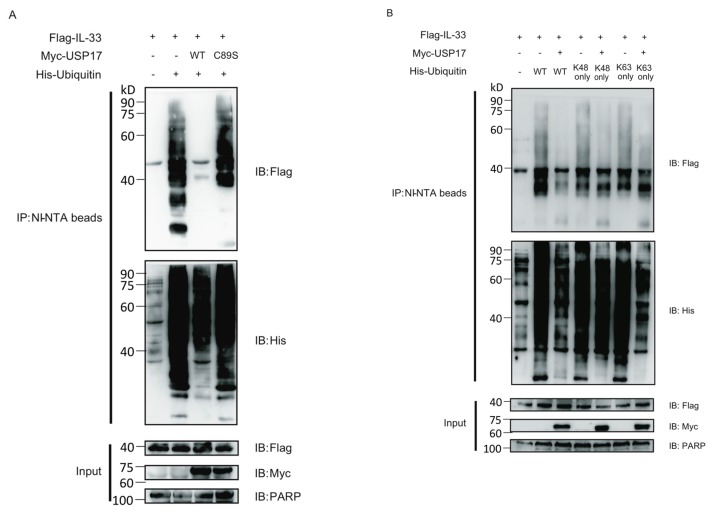
USP17 deubiquitinates IL-33. (**A**) HEK293T cells were transfected with Flag-IL-33, His-Ubi and Myc-USP17 or its enzymatically-inactive mutant C89S (USP17C89S). Cells were treated with 20 nM MG132 for 4 h and lysed. Ubiquitinated IL-33 was pulled-down with Ni-NTA beads. Immune blots were performed with the indicated antibodies; (**B**) HEK293T cells were transfected with Flag-IL-33, His-Ubi wild-type or K48/63 only and Myc-USP17. Cells were treated with 20 nM MG132 for 4 h and lysed. Ubiquitinated IL-33 was pulled-down with Ni-NTA beads. Immune blots were performed with the indicated antibodies. Data are representative of at least three independent experiments.

Since USP17 interacts with IL-33 and IL-33 can be post-translationally modified by polyubiquitination, we speculated that USP17 might deubiquitinate IL-33. To test our hypothesis, Flag-IL-33, His-ubiquitin and Myc-USP17 or its enzymatically-inactive mutant C89S (USP17C89S) were ectopically expressed in HEK293T cells, and then, ubiquitinated IL-33 was pulled down by Ni-NTA beads under denaturing conditions. We found that USP17 significantly decreased the polyubiquitination status of IL-33, while this effect was markedly reduced in the presence of its enzymatically-inactive mutant (Figure 3A). In order to determine which type of polyubiquitin chains were conjugated to IL-33, we performed a ubiquitin pull-down assay using ubiquitin mutants (K48 only, K63 only) that can only form K48- or K63-linked polyubiquitin chains. We found that both K48only and K63only polyubiquitin chains were linked to IL-33 to a degree that was comparable to the wild-type polyubiquitin chains. Additionally, USP17 could deubiquitinate IL-33 both at the K48 and at the K63 sites (Figure 3B). It appears that USP17 might affect both the stability and function of IL-33.

### 2.4. USP17 Stabilizes IL-33

**Figure 4:**
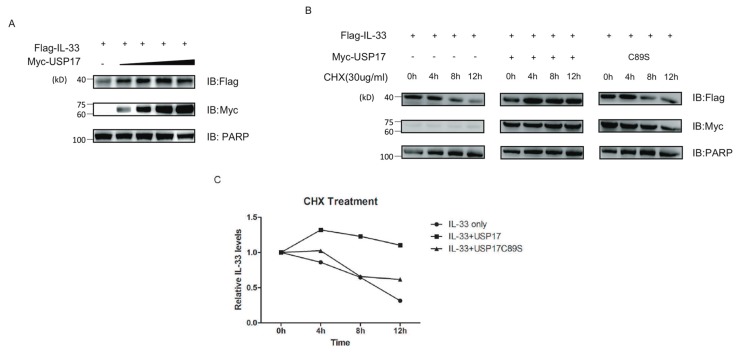
USP17 stabilizes IL-33. (**A**) HEK293T cells were transfected with Flag-IL-33 and increasing concentrations of Myc-USP17. Immune blots were performed with the indicated antibodies; (**B**) HEK293T cells were transfected with Flag-IL-33 and Myc-USP17 or its inactive mutant USP17C89S. Cells were treated with CHX (30 µg/mL) for different time points as indicated. Immune blots were performed with the indicated antibodies; (**C**) The protein levels of IL-33 were quantified by ImageJ software. All protein intensity values were normalized to 0 h protein values. Data are representative of at least three independent experiments.

To test whether the deubiquitinase USP17 could stabilize IL-33 at the protein level, 293T cells were co-transfected with Flag-tagged IL-33, increasing concentrations of Myc-tagged USP17 or empty vectors, and the protein levels were determined by immune blot. Interestingly, we found that USP17 stabilized IL-33 in a dose-dependent manner (Figure 4A). To further validate this conclusion, we ectopically co-expressed IL-33 and USP17 or its mutant C89S in the presence of the protein synthesis inhibitor cycloheximide (CHX) and detected the protein levels of IL-33 at the indicated time points by immune blot. Consistently, the over-expression of USP17 in the presence of CHX positively influenced the stability of the IL-33 protein (Figure 4B,C).

### 2.5. IL-33 Upregulates IL-13 by Chromatin Binding to the IL13 Gene Locus

**Figure 5:**
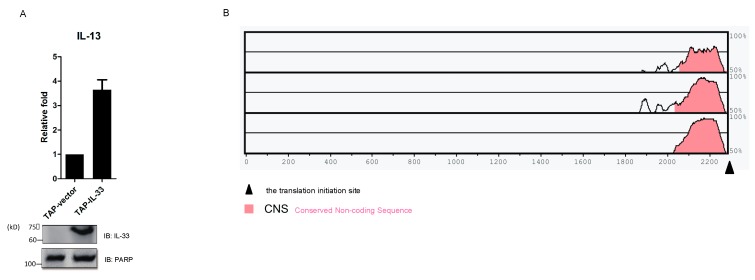

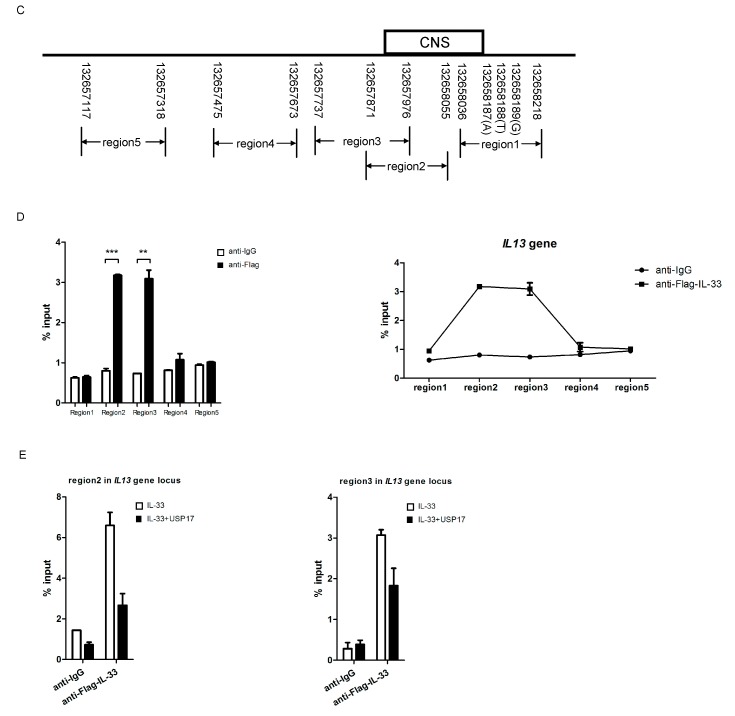
USP17 downregulates the chromatin binding of IL-33 to the CNS of *IL13* gene locus. (**A**) Total RNA was extracted from the TAP-IL-33 and TAP-vector stable cell lines reverse transcribed following standard procedures, and the mRNA level of IL-13 was detected by qPCR. Cell lysates were used to detect the expression of IL-33 by immune blot; (**B**) The human *IL13* genomic sequence was compared to house mouse, dog and cattle to find the conserved non-coding sequence (CNS) using the website [24]; (**C**) Five pairs of primers were designed based on the five regions, including the CNS, which localized before the translation initiation site (132658187 site); (**D**) HEK293T cells were transfected with Flag-IL-33. Cells were collected, and the rapid micro-chromatin immunoprecipitation assay (µChIP) was performed, as described in the Materials and Methods. Anti-Flag antibody and mouse IgG were used in this µChIP. ChIP and input samples were subjected to qPCR using the five pairs of primers described as above; (**E**) HEK293T cells were transfected with Flag-IL-33 and Myc-USP17 or an empty vector as a negative control. Cells were collected for µChIP analysis and then detected by qPCR using the two primers of region 2 and region 3 described as above. Data are representative of at least three independent experiments. *** *p* < 0.001. ** *p* < 0.01. Error bars represent mean ± SEM.

In order to figure out the target genes of IL-33, we detected the mRNA levels of several factors in a TAP-IL-33 stable cell line and found an increased mRNA level for IL-13 (Figure 5A). This suggested that *IL13* might be a downstream target of IL-33. To verify this hypothesis, we decided to perform a chromatin immunoprecipitation (ChIP) assay followed by quantitative PCR (qPCR) in 293T cells ectopically expressing Flag-IL-33. Therefore, we firstly compared the human *IL13* genomic sequence to the house mouse, dog and cattle ones and found a conserved non-coding sequence (CNS) before the translation initiation site (Figure 5B). Then, we designed five pairs of qPCR primers based on the five regions, including the CNS (Figure 5C). ChIP assay followed by qPCR revealed that IL-33 could directly bind to the CNS of the *IL13* gene locus and that the sequences corresponding to region 2 and region 3 might represent potential IL-33 binding sites (Figure 5D).

### 2.6. USP17 Downregulates the Chromatin Binding of IL-33 to the IL13 Gene Locus

Several studies showed that ubiquitination can regulate gene transcription, and our study showed that IL-33 can be polyubiquitinated; thus, we then investigated whether deubiquitinases could affect the nuclear function of IL-33. The ChIP assay was performed and showed that deubiquitinase USP17 could reduce the binding of IL-33 to both region 2 and region 3 (Figure 5E). Taken together, our data indicate that USP17 downregulates the chromatin binding of IL-33 to the CNS of the *IL13* gene locus.

## 3. Discussion

IL-33, like other members of the IL-1 family, holds a dual function, acting either as a classical cytokine via interaction with its receptor complex or exerting gene transcriptional regulatory functions in the nucleus. While the former has been thoroughly studied over a short period of time, its nuclear function has just attracted attention recently. One study suggests that the binding of IL-33 to the NF-κB p65 subunit in the nucleus could reduce p65-triggered gene expression [8], while another study suggests that nuclear IL-33 positively regulated *p65* transcription in endothelial cells by binding to the promoter region [9]. The specific nuclear targets and the biological effects of IL-33 still need to be elucidated in depth.

Ubiquitination represents a key molecular mechanism able to exert positive or negative regulatory effects on gene transcription and expression. In the past, studies aimed at understanding the function of ubiquitination were mainly focused on its effect on proteasome-mediated proteolysis, and only recently has research started to focus on its regulatory role on the function of target proteins [25]. Previously, it has been demonstrated that ubiquitin modification plays important roles in controlling gene transcription via modulating the activity of certain transcription factors or chromatin-associated proteins [11,26,27,28].

Being that ubiquitination is such a fundamental post-translational modification, we first attempted to evaluate the ubiquitination status of IL-33 and to explore the existence of any mechanism of deubiquitination that could regulate IL-33 stability or activity. The ubiquitin pull-down assay showed that polyubiquitin chains could be linked to IL-33 and that this process was reversible. After an accurate screening of potential DUBs, we found that IL-33 could interact with USP17, which was involved in the removal of polyubiquitin chains from IL-33. In particular, USP17 could stabilize the protein levels of IL-33 by deubiquitinating IL-33 at the K48 site. Furthermore, USP17 could also reverse the K63-linked ubiquitination of IL-33, which is associated with the modulation of protein function.

Next, we were interested in better elucidating the biological role of IL-33 and the consequences of IL-33 deubiquitination on its nuclear function. Previous studies have shown that IL-13 is an essential factor for the development of asthma and can be produced by airway epithelial cells [29,30]. IL-33 plays significant roles in the pathogenesis of asthma [31], and IL-13 can be induced by IL-33 via ST2 signaling. Thus, we wondered if IL-33 could regulate IL-13 not only as a cytokine, but also as a transcription factor. Consistent with our inference, the ChIP assay identified *IL13* as one of the target genes of IL-33 and revealed a crucial role for USP17 in impairing the binding activity of IL-33 to the CNS of *IL13* through deubiquitination.

There was still some limitation of our study. Although the post-translational modification of IL-33 was clearly studied, the nuclear function of IL-33 to regulate *IL13* was only studied in HEK293T cells. Further studies in a physiological environment should be performed. However, our study still, to some degree, illuminated the nuclear function of IL-33 and its regulation. Based on our findings, we will next perform ChIP analysis to figure out other target genes of IL-33 and to highlight the DNA-binding function of IL-33. These results will be of fundamental importance in order to understand the role of IL-33 in different kinds of allergic diseases.

To our knowledge, no data have been published, neither concerning the regulatory role of IL-33 on the *IL13* gene nor the regulatory network existing between USP17 and IL-33. In summary, our results point out a previously unknown role of IL-33 in the regulation of *IL13* and uncover a new mechanism of regulation of IL-33 via interaction with the USP17 deubiquitinase. Further studies are still needed to identify other nuclear functions of IL-33 and to highlight the physiological relevance of IL-33 modification by ubiquitination.

## 4. Materials and Methods

### 4.1. Cell Culture and Transfection

HEK293T cells were maintained in Dulbecco’s Modified Eagle’s Medium (DMEM, HyClone, Logen, UT, USA) containing 10% fetal bovine serum (FBS) at 37 °C and 5% CO_2_. Cells were transfected with expression plasmids using PEI (Polysciences, Warrington, FL, USA), according to the manufacturer’s instructions.

### 4.2. Antibodies and Reagents

The following antibodies were used: anti-Flag (M2, Sigma-Aldrich, Oakville, AB, Canada), anti-Myc (9E10, Santa Cruz Biotechnology, Dullas, TX, USA), anti-HA (F-7, Santa Cruz Biotechnology, Dullas, TX, USA), anti-tubulin (DM1A, Sigma, Oakville, AB, Canada), anti-PARP (436400, Invitrogen, New York, NY, USA), anti-mouse IgG HRP (85-18-8817-31, ebioscience, San Diego, CA, USA), anti-rabbit IgG HRP (85-18-8816-31, ebioscience, San Diego, CA, USA) and normal mouse IgG (sc-2025, Santa Cruz Biotechnology, Dullas, TX, USA). Protein A/G PLUS Agarose (sc-2003) was purchased from Santa Cruz Biotechnology, Dullas, TX, USA, and Ni-NTA beads (30210) from Qiagen, Duesseldorf, Germany. Cycloheximide (C7698-5G) was purchased from Sigma-Aldrich, Oakville, AB, Canada, and MG132 (CAS 133407-82-6) was from Merck, Darmstadt, Germany.

### 4.3. Plasmid Constructs and Stable Cell Line Construction

Expression plasmids encoding human IL-33, USP17 and ubiquitin, fused with Flag-, Myc- or His-tag, were constructed based on the pIRES2-puro vector by standard molecular biology techniques. Expression plasmids encoding human IL-33 fused with TAP-tag were constructed based on the pCDNA4/to-N-TAP vector. TAP-IL-33 and TAP-empty vector were transfected into HEK293T cells. Two days after transfection, zeocin was added to the culture for selecting cells stably expressing the transfected vectors. Immune blot and quantitative PCR were used for the identification of the TAP-IL-33 and TAP-vector stable cell line.

### 4.4. Immunoprecipitation and Immune Blot

For immunoprecipitation (IP) experiments, cells were lysed in RIPA buffer (50 mM Tris-HCl, pH 7.5; 150 mM NaCl; 1 mM EDTA; 1% NP-40; 0.25% NaDoc; 10% Glycerol) containing protease inhibitor cocktail (1:100, P8340; Sigma-Aldrich), 1 mM PMSF, 1 mM Na_4_VO_3_ and 1 mM NaF. Cell lysates were immunoprecipitated with the indicated antibodies for 2 h at 4 °C and then incubated with protein A/G PLUS Agarose for 1 h at 4 °C. The immunoprecipitates were washed with RIPA buffer (containing PMSF, Na_4_VO_3_ and NaF) three times and then analyzed by immune blot with the indicated antibodies.

### 4.5. Ubiquitin Pull-down Assay

Cells transfected with His-ubiquitin and Flag-IL-33 or Myc-USP17 were treated with 20 µM MG132 for 4 h and lysed in a pH 8.0 urea buffer (8 M urea, 100 mM Na_2_HPO_4_, 10 mM Tris (pH 8.0), 0.2% Triton X-100, 10 mM imidazole). For each sample, a part of the cell lysate was treated with Ni-NTA beads for 3 h at room temperature and used for the ubiquitin pull-down assay, while the rest was used as input. Beads were washed twice in pH 8.0 urea buffer, twice in pH 6.3 urea buffer (8 M urea, 100 mM Na_2_HPO_4_, 10 mM Tris (pH 6.3), 0.2% Triton X-100, 10 mM imidazole) and once in wash buffer (20 mM Tris (pH 8.0), 100 mM NaCl, 20% glycerol and 10 mM imidazole). Beads were then added to 2× loading buffer and boiled for 10 min. Samples were analyzed by immune blot with the indicated antibodies. The ubiquitination of IL-33 was detected with anti-Flag antibody.

### 4.6. Quantitative PCR

Total RNA was extracted from HEK293T cells using TRIzol reagent (Invitrogen, New York, NY, USA) and reverse transcribed using a reverse transcriptase kit (TaKaRa, Shiga, Japan) following the manufacturer’s instructions. Quantitative PCR was performed using SYBR green (TaKaRa, Shiga, Japan) on an ABI Prism 7500 system, and β-actin was used for normalization. The following primers were used for qPCR:

IL-33 forward: 5′-GAATCAGGTGACGGTGTTGA-3′;
IL-33 reverse: 5′-TCCAGGATCAGTCTTGCATTC3′;
β-actin forward: 5′-GGACTTCGAGCAAGAGATGG-3′;
β-actin reverse: 5′-AGCACTGTGTTGGCGTACAG-3′.

### 4.7. Rapid Micro-Chromatin Immunoprecipitation Assay on Quantitative PCR

The rapid micro-chromatin immunoprecipitation assay (µChIP) was performed as described in published protocols [32]. HEK293T cells were transfected with Flag-IL-33 and/or Myc-USP17 or its enzymatically-inactive mutant C89S (USP17C89S). Ten thousand cells were collected to be cross-linked with formaldehyde (1% final concentration). Cell lysates were sonicated under suitable conditions to produce chromatin fragments of ~500 bp (the range may be 200–1200 bp). The sheared chromatin fragments were immunoprecipitated with anti-Flag antibody or mouse anti-IgG as a negative control. The pulled down and purified DNA fragments were subjected to qPCR analysis. Primers for the conserved non-coding sequence before the translation initiation site in the *IL13* gene locus used for the qPCR experiments are as follows:

Region1-F: 5′-CGACACTGGATTTTCCACAA-3′;
Region1-R: 5′-GCCAACAGGAGAGGATTGAG-3′;
Region2-F: 5′-GGGTTGTCACAGGCAAAACT-3′;
Region2-R: 5′-TTGTGGAAAATCCAGTGTCG-3′;
Region3-F: 5′-ACTCCCCAAATTCCCATAGC-3′;
Region3-R: 5′-CCAAGATCTGGGTGGGTTTA-3′;
Region4-F: 5′-GTGAGACCCCATCTCCAAAA-3′;
Region4-R: 5′-GGCTTGCCTAATTCCTCCTT-3′;
Region5-F: 5′-GAGGGAAGAGCAGGAAAAGG-3′;
Region5-R: 5′-GCAGCCTTAGTCCAGGTCAG-3′;
Region6-F: 5′-GCTTCGAGTGTGGACAGAGA-3′;
Region6-R: 5′-TTCTCCCCTACCCACTTCCT-3′;
Region7-F: 5′-GATAAGGGGCGTTGACTCAC-3′;
Region7-R: 5′-CGTGTGACCCCTCTACGG-3′.

## Abbreviations

Cdc25A: cell division cycle 25A; ChIP: chromatin immunoprecipitation; CHX: cycloheximide; CNS: conserved non-coding sequence; DMEM: Dulbecco’s Modified Eagle’s Medium; DUB: deubiquitinase; E4F1: E4F transcription factor 1; FBS: fetal bovine serum; FBXL5: F-box and leucine-rich repeat protein 5 HectH9, HUWE1 HECT, UBA and WWE domain containing 1, E3 ubiquitin protein ligase; HEK293T cells: human embryonic kidney 293T cells; HTH: helix-turn-helix; H2A: histone H2A; H2B: histone H2B; IL-4: interleukin-4; IL-6: interleukin-6; IL-33: interleukin-33; IL-1RL1: interleukin 1 receptor-like 1; IL-1RAcP: IL-1 receptor accessory protein; IP: immunoprecipitation; JAMM: Josephins and JAB1/MPN/MOV34 metalloenzyme; MAP kinases: mitogen-activated protein kinase; NF-HEV: nuclear factor from high endothelial venules; NF-κB: nuclear factor kappa B; Ni-NTA: nickel-charged nitrilotriacetic acid; NK cells: natural killer cells; NKT cells: natural killer T cells; OUT: ovarian tumor proteases; PEI: polyethylenimine; PTM: post-translational modifications; RCE1: Ras-convertingenzyme1; SCF^Met30^: Skp1/Cul1/F-Box (SCF) complex containing the F-box protein Met30; SDS3: suppressor of defective silencing 3; Th2 lymphocytes: T helper 2 lymphocytes; Ub: ubiquitin; UCH: ubiquitin C-terminal hydrolase; USP: ubiquitin-specific protease; USP10: ubiquitin-specific protease 10; USP17: ubiquitin-specific protease 17; Snail1: snail family zinc finger 1.
